# Sequence-Based Classification Using Discriminatory Motif Feature Selection

**DOI:** 10.1371/journal.pone.0027382

**Published:** 2011-11-10

**Authors:** Hao Xiong, Daniel Capurso, Śaunak Sen, Mark R. Segal

**Affiliations:** 1 Department of Epidemiology and Biostatistics, University of California San Francisco, San Francisco, California, United States of America; 2 Department of Bioengineering and Therapeutic Sciences, University of California San Francisco, San Francisco, California, United States of America; The University of Queensland, Australia

## Abstract

Most existing methods for sequence-based classification use exhaustive feature generation, employing, for example, all 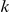-mer patterns. The motivation behind such (*enumerative*) approaches is to minimize the potential for overlooking important features. However, there are shortcomings to this strategy. First, practical constraints limit the scope of exhaustive feature generation to patterns of length 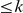, such that potentially important, longer (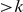) predictors are not considered. Second, features so generated exhibit strong dependencies, which can complicate understanding of derived classification rules. Third, and most importantly, numerous irrelevant features are created. These concerns can compromise prediction and interpretation. While remedies have been proposed, they tend to be problem-specific and not broadly applicable. Here, we develop a generally applicable methodology, and an attendant software pipeline, that is predicated on discriminatory motif finding. In addition to the traditional training and validation partitions, our framework entails a third level of data partitioning, a discovery partition. A discriminatory motif finder is used on sequences and associated class labels in the discovery partition to yield a (small) set of features. These features are then used as inputs to a classifier in the training partition. Finally, performance assessment occurs on the validation partition. Important attributes of our approach are its modularity (any discriminatory motif finder and any classifier can be deployed) and its universality (all data, including sequences that are unaligned and/or of unequal length, can be accommodated). We illustrate our approach on two nucleosome occupancy datasets and a protein solubility dataset, previously analyzed using enumerative feature generation. Our method achieves excellent performance results, with and without optimization of classifier tuning parameters. A Python pipeline implementing the approach is available at http://www.epibiostat.ucsf.edu/biostat/sen/dmfs/.

## Introduction

The abundance of genome-wide sequence data made possible by high-throughput technologies has sparked widespread interest in linking sequence information to biological phenotypes. For binary phenotypes, this amounts to sequence-based classification. For some such problems, the set of sequence features to be used as classifier inputs is evident from the problem context (e.g. genome-wide association studies [1], where the features are SNPs or haplotypes). In many other settings, however, this is not the case and the direct analysis of sequence data may not be possible. For example, when the phenotype of interest is the presence or absence of a molecular marker (such as a nucleosome), it may be necessary to first extract features (e.g., GC-content, 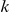-mer frequencies) from the underlying or nearby sequence prior to subsequent analysis. This process of coupling sequence-based feature extraction with downstream classification has been applied to several molecular genomic phenotypes, such as: CpG island methylation [2], escape from X inactivation [3], and nucleosome occupancy [4]. This approach has also been used in the *in silico* prediction of protein function (e.g., solubility [5]) from amino acid sequence [6].

Typically, such studies use *enumerative* feature generation, employing frequencies of all possible 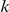-mers, thereby resulting in large numbers of candidate features. These features may be supplemented with additional features that, for example, capture information about local structure (e.g., DNA twist, DNA shear). The numbers of features generated for the studies mentioned above are sizeable: 1184 [2], 16788 [3], 2772 [4], and 16980 [5]. The logic behind enumerative feature generation is that having a wide-ranging suite of predictors ensures that nothing will be overlooked in downstream classification.

Support vector machines (SVMs) using specialized kernels, notably the spectrum kernel [7], and enumerative generation have been successfully deployed in several settings: classifying proteins [8], [9], splice sites [10], siRNA [11], and microRNAs [12], [13]. More sophisticated kernels, largely extending spectrum kernels, have advanced the scope and performance of such approaches [10], [14]–[16]. However, interpretation of feature importance is challenging for SVMs and, due to intrinsic dependencies, these difficulties are compounded for SVMs with enumeratively generated features [17]. Nonetheless, by using convex combinations of prescribed kernels, post-processing, and/or restriction to specific problems [17]–[20], important advances have been realized.

Besides interpretability, other concerns surround the use of enumerative feature generation. First, despite the seeming comprehensiveness of the approach, it may fail to generate key predictors because of feature length constraints. For example, when considering all 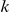-mers, the number of patterns grows as 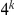 or 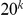 – depending on whether a nucleotide or amino acid alphabet is being utilized. Thus, 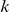 is restricted for computational reasons. For the four examples mentioned above [2]–[5], the respective limits are 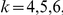 and 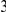. The implications of not being able to capture longer patterns are context dependent. Second, and perhaps more importantly, enumerative feature generation invariably creates a large number of irrelevant predictors or noise. As is well known [5], the presence of a large number of irrelevant features can degrade prediction and increase computation time.

We approach the sequence-based feature generation problem from a different tack. Rather than generating multitudes of largely irrelevant features only to discard the majority, we target elicitation of *a priori* informative features using a *discriminatory* motif finder. While loosely related work has appeared recently [21]–[24], these approaches are problem-specific. Here, we propose and implement a general-purpose framework for sequence-based classification. Of course, there is no free lunch: our approach requires another level of data partitioning (see Methods), since we use the phenotypes for feature selection.

Once discriminatory features are extracted, they are used as inputs for downstream classification. This is important for two reasons. First, classification enables a multivariate analysis. Though discriminatory motif finding algorithms yield ranked lists of candidate motifs, they are inherently univariate and do not evaluate potential interactions between motifs, an issue we return to in the Discussion. Further, other covariates, such as characterizations of genomic position (e.g., measures of evolutionary conservation, gene annotations), may be included as classifier inputs. Second, classification places motif significance assessment in a rigorous, well-developed inferential framework.

The paper is organized as follows. The next section outlines our proposed methodology, discriminatory motif-based feature selection (DMFS), and the attendant software implementation. The following section describes evaluation datasets and results comparing DMFS performance with enumerative approaches. The final section provides some concluding comments.

## Methods

We develop two-class classification rules where the predictors are sequences that need not be aligned or be of equal length. Additional covariates, if available, can be integrated, although we do not detail this aspect. We refer to data belonging to one class as *positive* (e.g. nucleosome occupied, or soluble proteins) and the other as *negative* (e.g. nucleosome unoccupied, or insoluble proteins).

**Figure 1:**
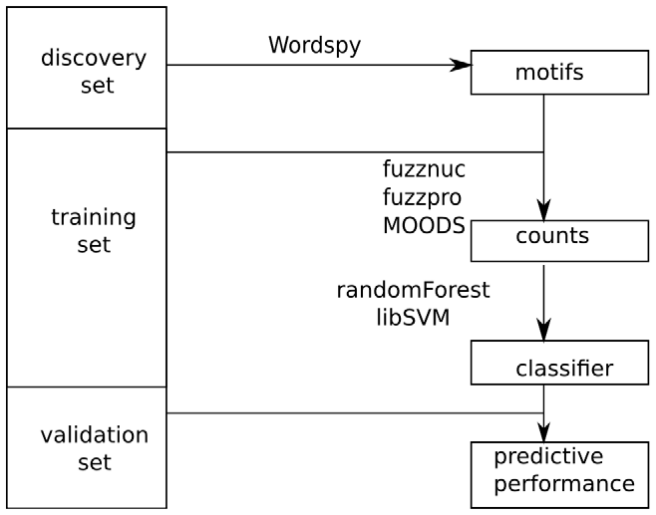
Illustrative diagram of data flow through the pipeline. Data is initially partitioned into discovery and classification sets. The classification set is further partitioned into training and validation sets. After WordSpy elicits motifs using the discovery set, fuzznuc or fuzzpro counts corresponding motif occurrences in the remaining data. The training data counts are used to train a classifier, while the validation data counts are used to determine performance (e.g. AUC) of the learned classifier.

The DMFS approach to classification proceeds as follows (see Figure 1 for a data flow diagram).

- *Partition step:* First, we partition the sequences and associated class labels into *discovery* and *classification* sets. This is an additional partitioning step required by DMFS.
- *Motif discovery step:* We use a discriminatory motif finder on the discovery set to find promising features (motifs). The motifs can be discrete or continuous, as represented by position weight matrices (PWMs).
- *Scoring step:* The selected features are used to score the classification set sequences (e.g., motif presence/absence or (weighted) motif counts).
- *Classification step:* Finally, the scores are used as inputs to perform conventional classification on the classification set. Traditional partitioning of data into training and testing sets is performed at this step.

Note that feature selection is performed using the discovery set only; discovery set data is withheld from classifier training and validation to avoid over-fitting and over-optimism due to data reuse.

DMFS can be customized to diverse data configurations by varying (a) the fraction of data used for feature discovery versus classification, (b) the discriminatory motif-finding algorithm and its attendant tuning parameters, (c) the scoring scheme for the classification set input sequences, and (d) the classification algorithm and its tuning parameters. Below, we elaborate on each step of our method, and the choices we made in our implementation.

### Partition step

The entire data set is randomly partitioned into discovery and classification sets, stratifying by class. Stratification preserves class proportions in the discovery and classification sets. The discovery fraction, 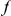, (proportion of the data used for motif discovery) should be large enough to yield meaningful motifs but not so large so as to degrade downstream classification. The optimal 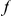 depends on the sample and class sizes, motif signal, motif complexity, and classifier learning rates, which are generally unknown. Thus prescribing an optimal 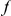 is difficult, but we found that 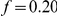 serves as a good starting point for reasonably large data sets. However, since 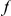 is a key tuning parameter of DMFS, sensitivity to different values should be explored.

### Motif discovery step

A discriminatory motif finder is applied on the discovery set to yield a set of promising sequence motifs. A wide variety of motif-finding methods are available including model-based [25]–[28], enumerative [29], [30], and dictionary-based [31], [32] approaches. Within each of these classes there are generative and discriminative methods, as well as hybrids thereof. Given our classification objectives we focus on discriminatory finders, as broadly advocated by Segal *et al.* [33] and Wang *et al.* [34].

We chose WordSpy [35] as the discriminatory motif finder for our pipeline implementation. We selected it because it has a readily-available, well developed, and robust implementation, and it exhibited superior performance in comparative studies: Wang and Zhang [35] compared 14 different motif-detection models on benchmark data composed of 56 curated datasets of sequences and motifs in 4 species [36], with WordSpy emerging as best. However, we emphasize that our pipeline is modular, and replacing WordSpy with alternate discriminatory motif finders is feasible.

WordSpy has a number of interrelated tuning parameters. For us, the most important is the maximum motif length, 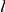. If long motifs drive the discrimination between positive and negative sequences, there is a premium on their elicitation. As explained in the Introduction, the exponential growth of 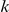-mers limits the extraction of long motifs via enumerative feature generation. However, even for WordSpy, finding long motifs is computationally demanding. For input nucleotide sequences of length 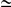 50, we have used maximal motif lengths in the range of 7–12. For protein sequences, the larger alphabet necessitates shorter lengths; for sequence lengths ranging from 15–1963 in the protein solubility dataset, we have used 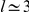
–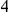.

### Scoring step

The motifs discovered from the discovery partition of sequences (above) are here used to create numerical vectors on the classification partition of sequences; these scores will later be used for training and validation. Our initial focus was on discrete motifs (as opposed to PWMs), these being the primary output of WordSpy. We use Fuzznuc or Fuzzpro (for DNA and protein sequences, respectively) from the EMBOSS suite of bioinformatics tools [37]. Fuzznuc/Fuzzpro efficiently count the number of motif occurrences in the input sequences, allowing for complementarity (strand) and a prescribed number of mismatches, 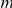. The latter is the sole tuning parameter of this step. As with the other parameters, universal prescriptions are misplaced, with 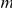 being dependent on 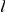. The default values we have employed are 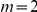 for nucleotide sequences (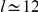) and 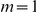 for protein sequences (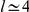). For scoring PWM motifs, we use MOODS [38]. Since motif degeneracies are embodied in the PWM formulation, there is no need for a mismatch parameter. Accordingly, the sole tuning parameter for MOODS is the pvalue, 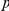, for determining if the total log odds score of a PWM at a sequence position is considered a match. MOODS slides the PWM (or a submatrix, using a lookahead algorithm) along both strands of the sequence and identifies scores corresponding to a pvalue 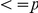. Whenever such a match is encountered, its score is added to a running sum, which then becomes a classification feature for that sequence.

### Classification step

After feature selection and scoring as described above, we obtain a dataset with numeric predictors corresponding to classification set sequences which, when used in conjunction with associated class labels, can be used for conventional classification analysis. Although there is a wealth of candidate classifiers, we chose to focus on two popular, flexible and complementary options for our pipeline: random forests (RF) [39] and support vector machines (SVM) [40]. For details, including classifier tuning, see Hastie *et al.* [41].

In brief, random forests construct an ensemble of classification trees and effect class assignments (for a given case) by a majority vote over the ensemble. Each tree in the forest is grown on a bootstrap sample of the data, and each split in an individual tree uses the best predictor/cut-point from a random subsample of the predictors. The purpose behind this deliberate injection of randomness is to de-correlate the trees in the ensemble, thereby yielding (prediction) variance gains when synthesizing over the ensemble. This strategy will be most successful when individual members of the ensemble result from an unstable classification technique; classification trees fit this criterion. Due to the bootstrap sampling (with replacement), approximately one third of the cases will be omitted from the construction of each tree. These samples are termed out-of-bag (OOB) and they can be used to obtain an unbiased estimate of classification accuracy, akin to cross-validation or sample splitting approaches.

There are numerous, inter-related measures of classification performance. We used the AUC (area under the receiver operator characteristic (ROC) curve) and classification accuracy, since these summaries were employed in the source enumerative analyses of the data considered subsequently. For random forests, the AUC is calculated using out-of-bag observations, while 10-fold cross-validation is used for SVMs. For random forests, the default parameters are: the number of candidate split variables set to one third of the total number of predictors, and growing a forest of 500 trees. For SVM, as implemented via LIBSVM [42], we used a Gaussian kernel with scale parameter (width) equal to 1/(number of features), and soft margin (which controls the error tolerance of the margin function) set to 1, the default set by LIBSVM.

### Software pipeline

We implemented our method in Python as a chain of executable programs that are naturally linked, the output of one stage being parsed by the next stage. The pipeline design is modular allowing different approaches and/or algorithms to be interchanged and tested. For example, the scoring stage can accommodate discrete or continuous motifs. The pipeline components can be run via a Python wrapper that, in addition to housekeeping, can parallelize different runs of the pipeline on machines with multicore/multi-processors. Further details are in the online documentation.

The principal tuning parameters available to the users are: the proportion of sample used for motif finding (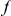), the maximum word length for motifs for WordSpy (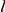), the number of mismatches tolerated for a motif to match a sequence (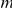), and the classification algorithm (RF or SVM).

The pipeline depends on WordSpy, the motif finding program; fuzznuc/fuzzpro, sequence analysis routines included in the EMBOSS package; MOODS, a suite of algorithms for PWM matching implemented in C++ with a Python interface; Biopython, a Python package providing biology-oriented computational tools; NumPy, a Python package for large multidimensional arrays; R/randomForest, an R library implementation of the random forest classifiers; and PyML a Python interface to SVM implementations. Our implementation supports a limited amount of parallelization; it can run with multiple threads on the same machine. Further details are in the software documentation. To our knowledge, all dependencies are met on Linux systems.

## Results

We applied DMFS to two distinct sequence-based classification problems in bioinformatics: (a) predicting nucleosome occupancy using nucleotide sequence, and (b) predicting protein solubility using amino acid sequence. For both problems, the goal was to classify sequences into one of two classes (nucleosome occupied or unoccupied, protein soluble or insoluble) based on the sequences alone.

Note that the two datasets represent very different problems with respect to experimental context, sequence number, sequence length, and sequence type. The nucleosome occupancy data was obtained from a single experiment or a set of related experiments, yielding DNA sequence of fixed length. The protein solubility data was curated from existing databases resulting in amino acid sequences of varying length. The sample size for the nucleosome occupancy data is 2000 while that for the protein solubility data is 17408.

For both the nucleosome occupancy and protein solubility data, we ran the pipeline on multiple random partitions of the data into *discovery sets* (see Methods). The reason for so doing is to avoid possible artifacts associated with ordering within data files and to provide robust performance assessments. Within each run, the data complementary to the randomly created discovery set serves as the *classification set* which, in turn, is partitioned into learning (training) and validation (testing) sets, either through use of cross-validation or, in the context of random forests, bootstrap resampling.

Previous analyses of these datasets employed enumerative feature generation, and are quoted below. We also undertook new analyses with the same enumeratively generated features, but using our SVM and random forest code and parameters. For both DMFS and enumerative feature generation approaches, we also utilized grid-search parameter tuning, although predictive performance at the default presets was not substantially different from that at optimal parameter settings.

### Nucleosome occupancy

The nucleosome, which consists of approximately 147-bp of DNA wrapped around an octamer of histones, is the basic unit of chromatin. The positioning (or phasing) of nucleosomes – via intrinsic DNA sequence preference and ATP-dependent chromatin remodelling complexes – can regulate gene expression by presenting or obscuring DNA regulatory elements [43]. Therefore, knowledge of nucleosome positioning is an important component to improved understanding of transcriptional control [44], [45]. While nucleosome *positioning* refers to the distribution of nucleosomes around a genomic position in a sample of cells [43], [46], nucleosome *occupancy* is a metric that indicates the coverage of a genomic position by nucleosomes in a sample of cells, regardless of the exact nucleosome start sites [46].

Human nucleosome occupancy data was obtained from Gupta *et al.* [4], who used primary data from two separate studies [44], [47]. The two datasets (labeled “Ozsolak” and “Dennis” after the respective lead authors) differ slightly in array design and in the methods used for ranking sequences. Both studies hybridized mononucleosomal DNA, as cleaved by MNase, onto tiling microarrays. For the Dennis dataset, DNA was extracted from an MDA-kb2 cell line. Log-ratio intensity measurements from custom microarrays, with probes spanning 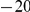 kb to 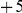 kb around transcription start sites of genes related to ATP-dependent chromatin remodeler response, were obtained. Gupta *et al.* [4] then procured positive and negative sequence sets by a ranking process applied to these intensities. In brief, this involved summing ranks for each locus (50-mer sequence) and each strand, and sorting following elimination of probes overlapping repetitive elements. Thresholding was then used to extract positive and negative sets, each containing 1000 50-mers. The Ozsolak dataset used DNA from seven cell lines and looked at 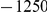 to 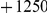 bases around the transcription start sites of human cancer-related and random genes. For this dataset, Gupta *et al.* 's ranking proceeded by first summarizing probe (50-mer sequence) intensities with a single value (mean, median or individual value depending on number of replicates), and then sorting the combined list. This list was traversed from top to bottom, and a probe was selected in the positive set when 5 of 7 cell types have been observed in the sorted list, as long as no probe within 50 basepairs had already been accepted into the positive set. Selection terminated following selection of 1000 sequences. The negative set, also comprising 1000 sequences, was similarly obtained by traversing from the bottom of the sorted list.

#### Ozsolak

For each of 40 randomly selected discovery sets, chosen with discovery fraction 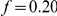 (see Methods), we applied our pipeline with maximum motif length 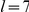, number of mismatches 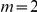 and both SVM and RF classifiers. Without tuning classifier parameters, the mean AUC for RF was 0.764 with a standard deviation (SD) of 0.0078. The mean AUC for SVM was 0.766 with SD 0.0084. Gupta *et al.* [4] reported a mean AUC of 0.737 on the entire Ozsolak dataset, which is slightly worse than the AUC we obtain. With classifier parameter tuning, the (optimal) AUCs increased to 0.768 for RF and 0.78 for SVM. We re-evaluated enumerative feature generation using our pipeline classifiers and the same grid-search for tuning parameter optimization. The AUC was 0.79 for RF and 0.8 for SVM. In general, the pipeline and the enumerative methods generate similar performances. The results are presented in Table 1.

**Table 1:** Nucleosome occupancy data.

Dataset	DMFS Default		DMFS Tuned		Reported	Enumerative	
	SVM	RF	SVM	RF	SVM	SVM	RF
Dennis	0.908	0.902	0.91	0.905	0.908	0.92	0.918
Ozsolak	0.766	0.764	0.78	0.768	0.737	0.8	0.79

Mean AUCs for the nucleosome occupancy datasets and approaches as described in the text. Reported values are from Gupta *et al.* [4]. The DMFS pipeline results are stable with small standard deviations as determined by 40 runs with random data partitioning: Dennis data with (a) default parameter settings: 0.0055 (SVM) and 0.0036 (RF), and (b) tuned parameter settings: 0.0048 (SVM) and 0.0041 (RF); Ozsolak data with (a) default parameter settings: 0.0084 (SVM) and 0.0078 (RF), and (b) tuned parameter settings: 0.011 (SVM) and 0.0086 (RF).

**Figure 2:**
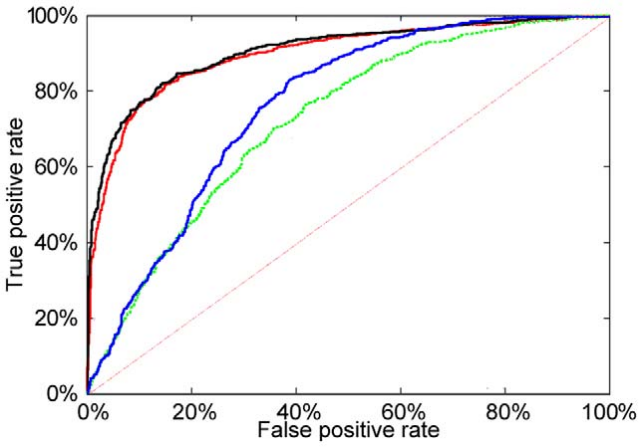
ROC curves from DMFS and enumerative methods for the nucleosome occupancy datasets. The red and green curves are from Gupta *et al.* [4] for the Dennis and Ozsolak data respectively. The black and blue curves are from the DMFS method for the Dennis and Ozsolak data respectively. For both datasets, the DMFS ROC curve is approximately equal to the ROC curve using enumerative feature generation. This figure was created by manipulating Figure 1 of Gupta *et al.* [4] in GIMP. The DMFS ROC curves are relative stable. As the false positive rate ranges from 10% to 90% the true positive rate standard deviations have range 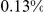 to 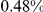 for the Dennis data and 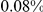 to 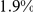 for the Ozsolak data.

From the 40 runs of our pipeline at default parameter values, we picked one at random and plotted the ROC curve of the random forest classifier, and superimposed the plot onto the average ROC curve for the same dataset from Gupta *et al.* [4] (Figure 2) The blue curve plots the ROC for DMFS which is at least comparable to Gupta *et al.* 's result shown in green.

In addition, we compared the filtered motifs generated in our pipeline with significant features of nucleosome occupancy identified in Tillo and Hughes [48] and Lee *et al.* [49]. Among features of length four, AAAA/TTTT is the most significant, and we found it occurring as part of motifs in 36 runs out of a total of 40 runs. Among other significant features, GAAA/TTTC is in 40 runs, and AGAA in 34. The full list is in Table 2.

**Table 2:** DMFS pipeline recovery of previously identified motifs.

Reported motif	Dennis	Ozsolak
AAAA/TTTT	37	36
AAAT	26	28
AATA	7	6
ATAA	5	4
GAAA	37	38
ATTA	2	8
TATA	1	1
AATT	6	19
ATAT	2	6
AGAA	37	34
AAGT	28	22
CGCG	4	0
TGGA	32	39
GCGC	9	1
CCCG/CGGG	13	4
CGGC/GCCG	4	8
GAAA/TTTC	40	40
CCGC/GCGG	10	4

Here we list motifs identified by Tillo and Hughes [48] and Lee *et al.* [49] and the number of times these motifs were identified by the DMFS pipeline. Structure related features are omitted, as are transcription binding start sites and features with zero weights. We ran the DMFS pipeline 40 times, with random data partitioning, and counted the number of times each previously identified motif occurred. According to Tillo and Hughes the most discriminative motif is the 4-mer AAAA/TTTT, which emerged in almost every run.

#### Dennis

The same configuration and parameters employed in analyzing the Ozsolak data were again used. The mean AUC for RF was 0.902 with SD 0.0036. The mean AUC for SVM was 0.908 with SD 0.0055. This matches the results of Gupta *et al.* [4] who also reported an AUC of 0.908. Tuning classifier parameters again produced only incremental improvements, with AUCs increasing to 0.905 for RF and 0.91 for SVM. As above, we re-applied enumerative methods using pipeline classifiers and grid search tuning. This yields AUCs of 0.918 and 0.92 for RF and SVM respectively, as shown in Table 1.

As with the Ozsolak example, we superposed ROC curves; see Figure 2. The DMFS curve (black) is once again at least comparable to the Gupta *et al.* [4] curve (red). Finally, we also compared the filtered motifs generated with features in Tillo and Hughes [48] as well as Lee *et al.* [49]. AAAA/TTTT was in 37 out of 40 runs, while GAAA/TTTC is in 40, and AGAA in 37. The full list is in Table 2.

### Protein solubility

The protein solubility data used derive from a carefully curated compilation that yielded a large, non-redundant, and balanced dataset [5]. Dataset creation proceeded by initially pooling *E.coli* protein sequences from the Protein Data Bank [50], SwissProt database [51], TargetDB [52], and Idicula-Thomas and Balaji [53]. Redundant sequences were then eliminated using BLASTCLUST [54], and the resulting set further reduced to achieve class balance finally yielding 8704 “soluble” and 8704 “insoluble” sequences. The soluble proteins, whose lengths range from 12 to 1901 basepairs, have a mean sequence length of 231.85 , a median length of 166; and the insoluble portion has a range of 21 to 1963 basepairs with a mean length 277.4 and a median of 229.

For each of 20 randomly selected discovery sets, chosen with discovery fraction 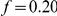, we applied our pipeline with maximum motif length 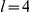, number of mismatches 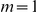 and both SVM and RF classifiers. The restriction to tetramers as maximum motif length reflects the increased complexity of the amino acid (versus nucleotide) alphabet and contrasts with the specification of heptamers (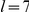) for the nucleosome occupancy problems.

With default classifier parameter values the RF mean accuracy was 0.61 with SD 0.0048 and the SVM mean accuracy was 0.62 with SD 0.0060. Magnan *et al.* [5] reported an accuracy of 0.548 using SVM and all trimer frequencies, use of tetramers being precluded since 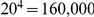 features proved too computationally demanding for effective parameter-tuning. Tuning of classifier parameters via grid search yielded accuracies of 0.645 and 0.63 for RF and SVM respectively. We also re-fitted the enumerative approach, generating all possible amino acids sequences of lengths 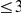. With SVM parameter tuning we obtained an accuracy of 0.63. Full results are presented in Table 3.

**Table 3:** Protein solubility data.

DMFS Default		DMFS Tuned		Enumerative		Reported	
SVM	0.62	SVM	0.63	SVM ( 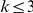 )	0.63	1-mer (SVM)	0.644
RF	0.61	RF	0.645	RF ( 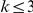 )	0.64	2-mer (SVM)	0.597
						3-mer (SVM)	0.548

Protein solubility data accuracies for default and tuned parameters settings, as well as for reported and eumerative methods. Reported values are from Magnan *et al.* [5]. The DMFS pipeline results are stable with small standard deviations as determined by 20 runs with random data partitioning: (a) default parameter settings: 0.006 (SVM) and 0.0048 (RF), and (b) tuned parameter settings: 0.0052 (SVM) and 0.0049 (RF).

## Discussion

We have presented and implemented a new method for sequence-based classification. We evaluated its performance on two nucleosome occupancy datasets and a protein solubility dataset. DMFS achieved performance comparable to previously published results on the same data using enumerative feature generation. This conclusion did not change on re-evaluating enumerative methods by refitting using our pipeline classifiers, both with and without classifier parameter tuning. These findings demonstrate the potential of DMFS as a general purpose method for sequence-based classification.

In this paper, we emphasized the prediction performance of DMFS. Additionally, we demonstrated that DMFS is effective at identifying important sequence features, as illustrated by its precursor [55]. We note that the interpretability of analyses using enumeratively generated features may be improved. For example, techniques that provide feature ranking, such as random forests and gradient boosting [56], could be utilized. However, current efforts toward feature interpretation for enumerative methods (e.g. POIMs [17]) have focussed on SVMs and, arguably, have thus required restrictions on kernels and input sequences.

It is important to recognize that we do not claim that DMFS constitutes a superior approach to enumerative feature generation. The performance results presented herein show comparable performance. While we have identified some putative advantages of DMFS (ability to accommodate longer features, elimination of noise features), there are complementary strengths of enumerative methods. These assets include: (i) the ability to recover important *feature interactions* in the absence of main effects, and (ii) the sample size benefit of requiring only two (as opposed to three) levels of data partitioning. With regard to (i), the extent to which there are features that operate exclusively interactively is a subject of long standing debate.

Our current implementation of DMFS uses particular choices of motif finder (WordSpy), motif scorers (fuzznuc, fuzzproo, MOODS), and classifiers (RF, and SVM). It is important to distinguish the *method* from its *realization* in our Python implementation. DMFS can use other motif finders, scorers, or classifiers. For example, in some applications it may be more fruitful to use a different classifier (such as gradient boosting) or a different motif finder (such as DEME [57]). The data flow would be exactly as in Figure 1, but with different plugins.

In developing our computational pipeline, we opted to emphasize modularity, and this results in some limitations. Allowing easy swapping of differing motif-finders and classifiers mandated certain design choices. For one, we did not reserve a validation set at the beginning of the pipeline, because resampling based methods such as bagging and random forests do not require such a set.

The modularity of our pipeline can be seen in the different tools we used in the scoring step. It uses Fuzznuc/Fuzzpro for discrete motifs, and MOODS [38] for continuous motifs as represented via position weight matrices, all of which are speed and memory efficient. As MOODS is already integrated with BioPython, incorporation into our Python pipeline was straightforward. While the more recently released standalone PWM scoring tool FIMO [58], which is also fast, possesses many desirable add-on features (e.g., multiple testing corrections, a variety of output formats), these are not needed for our purposes. More importantly, the absence of a Python interface would make for a much more involved integration, with likely run-time penalties.

We opted not to formally integrate grid-search based tuning of hyperparameters into the pipeline for practical reasons. Meaningful ranges for many such parameters (e.g. maximal motif lengths and number of mismatches) are both problem-specific and inter-related. Similarly, discovery set proportion is influenced by sample size and class proportions. Proliferation in the number of parameter combinations required for context-free grid-search makes such optimization highly computationally intensive. Our pipeline design readily supports such exploration and tuning via wrappers.

Future work on DMFS will proceed in several directions. We will pursue a systematic study of tuning parameter specification, hoping to refine the proffered guidelines. More ambitious is extension of the framework to phenotypes beyond the two class categorical outcomes examined here. While multi-category outcomes could be addressed by adopting the one-against-all stratagem, both synthesis and implementation issues will require further development. Finally, direct handling of multi-categories, as well as continuous outcomes, will mandate an entirely new approach to discriminatory motif finding.

## References

[1] Hirschhorn J, Daly M (2005). Genome-wide association studies for common diseases and complex traits.. Nature Reviews Genetics.

[2] Bock C, Paulsen M, Tierling S, Mikeska T, Lengauer T (2006). CpG island methylation in human lymphocytes is highly correlated with DNA sequence, repeats, and predicted DNA structure.. PLoS Genet.

[3] Wang Z, Willard HF, Mukherjee S, Furey TS (2006). Evidence of inuence of genomic dna sequence on human x chromosome inactivation.. PLoS Comput Biol.

[4] Gupta S, Dennis J, Thurman RE, Kingston R, Stamatoyannopoulos JA (2008). Predicting human nucleosome occupancy from primary sequence.. PLoS Computational Biology.

[5] Magnan CN, Randall A, Baldi P (2009). SOLpro: accurate sequence-based prediction of protein solubility.. Bioinformatics.

[6] Sleator RD, Walsh P (2010). An overview of in silico protein function prediction.. Archives of Microbiology.

[7] Leslie C, Eskin E, Noble WS (2002). The spectrum kernel: a string kernel for SVM protein classification.. Pacific Symposium on Biocomputing Pacific Symposium on Biocomputing.

[8] Leslie C, Kuang R (2004). Fast string kernels using inexact matching for protein sequences.. J Mach Learn Res.

[9] Shamim MTA, Anwaruddin M, Nagarajaram H (2007). Support vector machine-based classification of protein folds using the structural properties of amino acid residues and amino acid residue pairs.. Bioinformatics.

[10] Sonnenburg S, Schweikert G, Philips P, Behr J, Ratsch G (2007). Accurate splice site prediction using support vector machines.. BMC Bioinformatics.

[11] Teramoto R, Aoki M, Kimura T, Kanaoka M (2005). Prediction of siRNA functionality using generalized string kernel and support vector machine.. FEBS Letters.

[12] Xue C, Li F, He T, Liu G, Li Y (2005). Classification of real and pseudo microRNA precursors using local structure-sequence features and support vector machine.. BMC Bioinformatics.

[13] Ng KLS, Mishra SK (2007). De novo SVM classification of precursor microRNAs from genomic pseudo hairpins using global and intrinsic folding measures.. Bioinformatics.

[14] Leslie C, Eskin E, Noble W (2003). Mismatch string kernels for SVM protein classification.. http://citeseerx.ist.psu.edu/viewdoc/summary?doi=10.1.1.58.4737.

[15] Leslie CS, Eskin E, Cohen A, Weston J, Noble WS (2004). Mismatch string kernels for discriminative protein classification.. Bioinformatics.

[16] Ratsch G, Sonnenburg S, Scholkopf B (2005). RASE: recognition of alternatively spliced exons in c.elegans.. Bioinformatics.

[17] Sonnenburg S, Zien A, Philips P, Rtsch G (2008). POIMs: positional oligomer importance matricesunderstanding support vector machine-based signal detectors.. Bioinformatics.

[18] Sonnenburg S, Rtsch G, Schfer C (2005). Learning interpretable SVMs for biological sequence classification.. BMC BIOINFORMATICS.

[19] Sonnenburg S, Rieck K, Ida FF, Rtsch G (2007). Large scale learning with string kernels.. LARGE SCALE KERNEL MACHINES.

[20] Schultheiss S, Busch W, Lohmann J, Kohlbacher O, Ratsch G (2009). Kirmes: kernel-based identification of regulatory modules in euchromatic sequences.. BMC Bioinformatics.

[21] Lin Th, Murphy RF, Bar-Joseph Z (2011). Discriminative motif finding for predicting protein subcellular localization.. IEEE/ACM Transactions on Computational Biology and Bioinformatics.

[22] Ma PC, Chan KC (2010). Discovering interesting motif-sets for multi-class protein sequence classification.. Journal of Computational Biology: A Journal of Computational Molecular Cell Biology.

[23] Rao A, Hero AO, States DJ, Engel JD (2007). Motif discovery in tissue-specific regulatory sequences using directed information.. EURASIP J Bioinformatics Syst Biol.

[24] Vens C, Rosso M, Danchin EGJ (2011). Identifying discriminative classification-based motifs in biological sequences.. Bioinformatics.

[25] Lawrence C, Altschul S, Boguski M, Liu J, Neuwald A (1993). Detecting subtle sequence signals - A Gibbs sampling strategy for multiple alignment.. Science.

[26] Bailey T, Elkan C (1995). Unsupervised learning of multiple motifs in biopolymers using expectation maximization.. Machine Learning.

[27] Hertz G, Stormo G (1999). Identifying DNA and protein patterns with statistically significant alignments of multiple sequences.. Bioinformatics.

[28] Hughes J, Estep P, Tavazoie S, Church G (2000). Computational identification of cis-regulatory elements associated with groups of functionally related genes in Saccharomyces cerevisiae.. Journal of Molecular Biology.

[29] Van Helden J, Andre B, Collado-Vides J (1998). Extracting regulatory sites from the upstream region of yeast genes by computational analysis of oligonucleotide frequencies1.. Journal of Molecular Biology.

[30] Sinha S, Tompa M (2003). YMF: A program for discovery of novel transcription factor binding sites by statistical overrepresentation.. Nucleic Acids Research.

[31] Bussemaker H, Li H, Siggia E (2000). Building a dictionary for genomes: Identification of presumptive regulatory sites by statistical analysis.. Proceedings of the National Academy of Sciences of the United States of America.

[32] Gupta M, Liu J (2003). Discovery of conserved sequence patterns using a stochastic dictionary model.. Journal of the American Statistical Association.

[33] Segal E, Barash Y, Simon I, Friedman N, Koller D (2002). From promoter sequence to expression: a probabilistic framework..

[34] Wang G, Yu T, Zhang W (2005). WordSpy: identifying transcription factor binding motifs by building a dictionary and learning a grammar.. Nucleic Acids Research.

[35] Wang G, Zhang W (2006). A steganalysis-based approach to comprehensive identification and characterization of functional regulatory elements.. Genome Biol.

[36] Tompa M, Li N, Bailey TL, Church GM, Moor BD (2005). Assessing computational tools for the discovery of transcription factor binding sites.. Nature Biotechnology.

[37] Rice P, Longden I, Bleasby A (2000). EMBOSS: the European Molecular Biology Open Software Suite.. Trends Genet.

[38] Korhonen J, Martinmki P, Pizzi C, Rastas P, Ukkonen E (2009). Moods: fast search for position weight matrix matches in dna sequences.. Bioinformatics.

[39] Breiman L (2001). Random forests.. Machine Learning.

[40] Cristianini N, Shawe-Taylor J (2000). An Introduction to Support Vector Machines.

[41] Hastie T, Tibshirani R, Friedman J (2009). The elements of statistical learning: data mining, inference and prediction.

[42] Fan R, Chen P, Lin C (2005). Working set selection using second order information for training support vector machines.. The Journal of Machine Learning Research.

[43] Jiang C, Pugh B (2009). Nucleosome positioning and gene regulation: advances through genomics.. Nature Reviews Genetics.

[44] Dennis JH, Fan HY, Reynolds SM, Yuan G, Meldrim JC (2007). Independent and complementary methods for large-scale structural analysis of mammalian chromatin.. Genome Research.

[45] Kornberg R, Lorch Y (2002). Chromatin and transcription: where do we go from here.. Current Opinion Genetics Development.

[46] Kaplan N, Hughes T, Lieb J, Widom J, Segal E (2010). Contributions of histone sequence preferences to nucleosome organization: proposed definitions and methodology.. Genome Biology.

[47] Ozsolak F, Song JS, Liu XS, Fisher DE (2007). High-throughput mapping of the chromatin structure of human promoters.. Nature Biotechnology.

[48] Tillo D, Hughes T (2009). G+C content dominates intrinsic nucleosome occupancy.. BMC Bioinformatics.

[49] Lee W, Tillo D, Bray N, Morse RH, Davis RW (2007). A high-resolution atlas of nucleosome occupancy in yeast.. Nat Genet.

[50] Berman HM, Westbrook J, Feng Z, Gilliland G, Bhat TN (2000). The protein data bank.. Nucleic Acids Research.

[51] The UniProt Consortium (2007). The universal protein resource (UniProt).. Nucleic Acids Research.

[52] Chen L, Oughtred R, Berman HM, Westbrook J (2004). Targetdb: a target registration database for structural genomics projects.. Bioinformatics.

[53] Idicula-Thomas S, Balaji PV (April 2005). Understanding the relationship between the primary structure of proteins and their amyloidogenic propensity: clues from inclusion body formation.. Protein Engineering Design and Selection.

[54] Altschul SF, Madden TL, Schffer AA, Zhang J, Zhang Z (1997). Gapped blast and psi-blast: a new generation of protein database search programs.. Nucleic Acids Research.

[55] Segal M (2008). Re-cracking the nucleosome positioning code.. Statstical Applications in Genetcs and Molecular Biology.

[56] Friedman JH (2011). Greedy function approximation: A gradient boosting machine.. The Annals of Statistics.

[57] Redhead E, Bailey T (2007). Discriminative motif discovery in DNA and protein sequences using the DEME algorithm.. BMC Bioinformatics.

[58] Grant CE, Bailey TL, Noble WS (2011). Fimo: scanning for occurrences of a given motif.. Bioinformatics.

